# Eyelid control during an eye examination

**Published:** 2009-12

**Authors:** Sue Stevens

**Affiliations:** Former Nurse Advisor, *Community Eye Health Journal*, International Centre for Eye Health, London School of Hygiene and Tropical Medicine, Keppel Street, London WC1E 7HT, UK.

**Figure:**
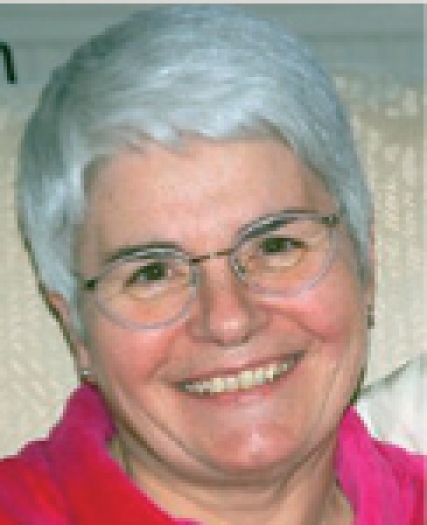


**Before performing any eye procedure**

- **Wash your hands** (and afterwards too)
- **Position the patient comfortably** with head supported
- **Avoid distraction** for yourself and the patient
- Ensure **good lighting**
- Always **explain to the patient** (and any companion, if appropriate) what you are going to do.

**Reasons for eyelid control during eye examination**

- to provide a good view of the eyeball for the examiner
- to avoid unnecessary discomfort for the patient

**Remember!**

**It is important to be very gentle at all times, in particular when an injured, painful, or postoperative eye is being examined. To do otherwise may cause further problems. Eyelid control is very important!**

**Preparation**

Position the patient comfortably. Depending on the circumstances, this may be:

- lying down with his or her head on a pillow
- sitting down with his or her head resting against a wall or headrest, or with the head supported by the hands of an assistant (Figure 1)
- sitting down at a slit lamp with head supported on the chin rest.

**Figure:**
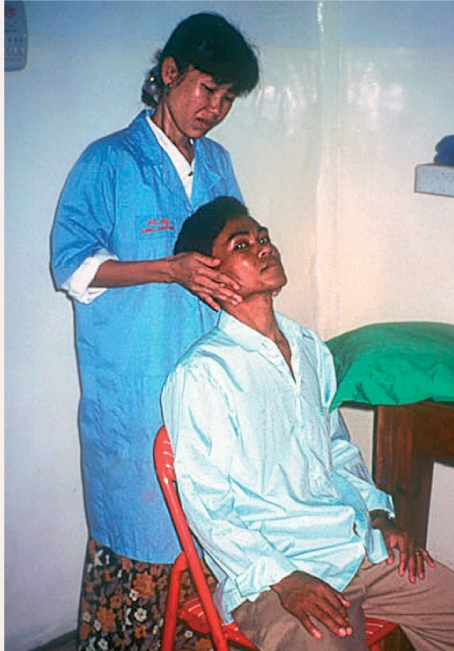
Figure 1

**Method**

- Ask the patient to **look up** and hold this gaze
- With the index finger, **gently and slowly** pull down the lower eyelid

**This position will enable a good view of the lower eyelid margin and lower eyeball** (Figure 2).

**Figure:**
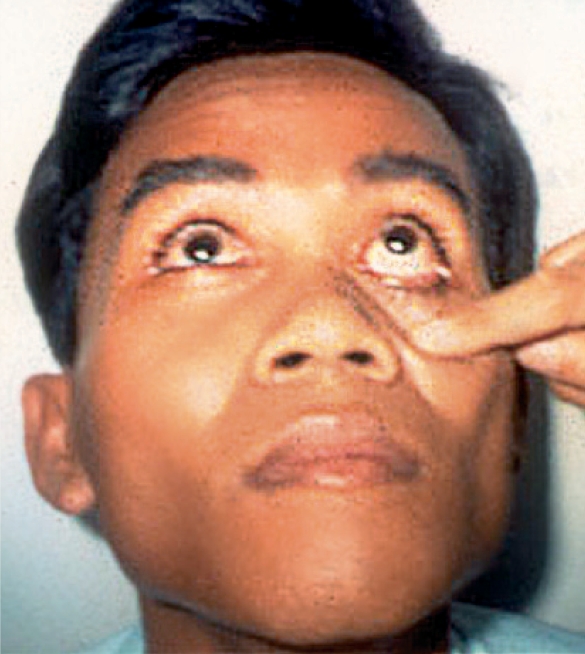
Figure 2

- When examination of this area is complete, **gently and slowly** remove the index finger and allow the patient to close the eyes for a few seconds
- Ask the patient to **look down** and to hold this gaze
- With the tip of the thumb, **gently and slowly** touch the top eyelid midway between the eyelid margin and the eyebrow (Figure 3) - **do not exert any pressure!**
- Ease the eyelid up, **gently and slowly**, against the bony orbital rim

**Figure:**
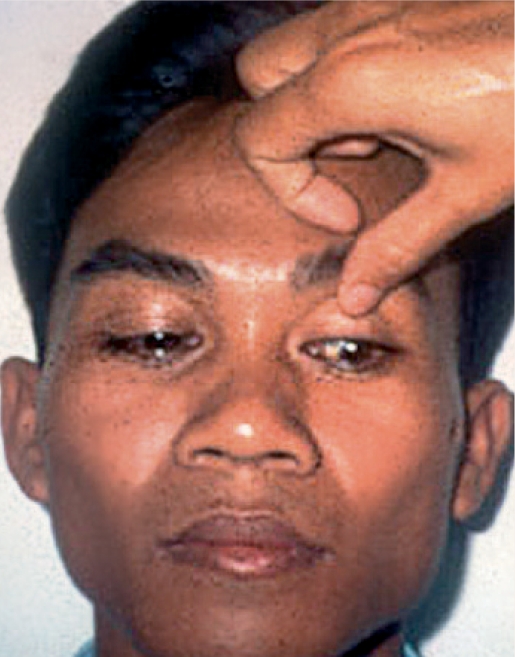
Figure 3

**This position will enable a good view of the upper eyelid margin and the upper eyeball** (Figure 4).

**Figure:**
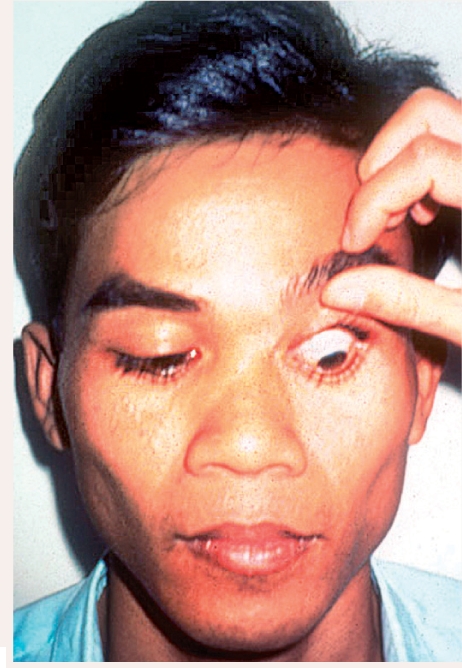
Figure 4

- When examination of this area is complete, **gently and slowly** remove the thumb and allow the patient to close the eyes
- Tell the patient when the examination has ended.

**IMPORTANT!** These principles should be followed **every time** and by **every examiner**.

